# Changes in the Fatty Acid Profile of Lactating Women Living in Poland—A Comparison with the Fatty Acid Profile of Selected Infant Formulas

**DOI:** 10.3390/nu16152411

**Published:** 2024-07-25

**Authors:** Aleksandra Purkiewicz, Renata Pietrzak-Fiećko

**Affiliations:** Department of Commodity Science and Food Analysis, Faculty of Food Science, University of Warmia and Mazury, Plac Cieszyński 1, 10-718 Olsztyn, Poland; renap@uwm.edu.pl

**Keywords:** human milk composition, food for infants, fatty acid profile, breastfeeding, lactation period

## Abstract

The present study examined the fatty acid content of human milk from Polish women living in the Warmia and Mazury region with regard to different lactation periods and compared it with the fatty acid content of selected infant formulas. The analysis included samples of breast milk—colostrum (*n* = 21), transitional milk (*n* = 26), and mature milk (*n* = 22). Fat was extracted using the Rose-Gottlieb method, and the fatty acid profile was determined by gas chromatography with a flame ionization detector (FID). The proportion of SFAs (saturated fatty acids) > MUFAs (monounsaturated fatty acids) > PUFAs (polyunsaturated fatty acids) was determined in each fraction of breast milk and infant formula. Palmitic, oleic, and linoleic acids predominated in breast milk and infant formulas. Colostrum contained lower contents of selected SFAs (caprylic, capric, lauric) and higher contents of selected MUFAs (ercucic) and PUFAs (arachidonic and docosahexaenoic) (*p* < 0.05) relative to transitional and mature milk. Infant formulas were distinguished from human milk in terms of their SFA (caproic, caprylic, lauric, arachidic), MUFA (oleic), and PUFA (linoleic, α-linoleic) content. It should be noted that infant formulas contained significantly lower trans fatty acid (TFA) content—more than thirty-six and more than nineteen times lower than in human milk. Furthermore, human milk contained branched-chain fatty acids (BCFAs) at 0.23–0.28%, while infant formulas contained only trace amounts of these acids. The average ratio of *n*-6 to *n*-3 fatty acids for human milk was 6.59:1 and was close to the worldwide ratio of 6.53 ± 1.72:1. Both principal component analysis (PCA) and cluster analysis (CA) indicated significant differences in the fatty acid profile relative to lactation and a different profile of infant formulas relative to breast milk.

## 1. Introduction

Human breast milk is the optimal dietary source of nutrients for infants. Its composition is adapted to the needs of the child in terms of all the nutrients, bioactive compounds, as well as immune factors that are essential for the development of the young organism [1]. The infant period is critical for the proper development of organs and tissues; therefore, nutrition is crucial at this stage. According to the recommendations of the World Health Organization (WHO), it is recommended that breast milk should be the only source of nutrition for the first six months of a child’s life. Natural feeding should be continued until 24 months of age, with the addition of appropriate complementary foods [2].

The predominant component of breast milk is water (87–88%), and the remaining components include solids: carbohydrates (7%), lipids (3.8%), and proteins (1%) [3]. The most notable of these are lipids, which provide about 50% of the baby’s energy during infancy [2]. During lactation, the fat content of breast milk can vary from 3.5 to 4.5%. Breast milk fat consists of 98% triacylglycerols (TAGs), with the remainder consisting of phospholipids and cholesterol [4]. The dominant fatty acids in breast milk are saturated fatty acids (SFAs) and monounsaturated fatty acids (MUFAs), followed by omega-3 (*n*-3) and omega-6 (*n*-6) polyunsaturated fatty acids (PUFAs) [5]. Half of the fatty acids in breast milk consist of SFAs, with palmitic acid (C16:0) being the dominant one, accounting for about 23% of all fatty acids. In contrast, the highest proportion in breast milk is oleic acid (C18:1 *n*-9), accounting for about 36% of the proportion of fatty acids [6].

Essential fatty acids, including linoleic acid (18:2 *n*-6; LA) and α-linolenic acid (C18:3 *n*-3; ALA), are particularly important in infant development. These acids are precursors to physiologically important polyunsaturated metabolites, including arachidonic acid (C20:4 *n*-6; AA), eicosapentaenoic acid (C20:5 *n*-3; EPA) as well as docosahexaenoic acid (C22:6 *n*-3; DHA). Particular focus in infant development should be on DHA, which can accumulate in the membrane lipids of the brain and retina during the perinatal period, where it is responsible for the formation of visual acuity and the motor system of newborns [7]. It has been indicated that the intake of long-chain polyunsaturated fatty acids (LCPUFAs) in the diet is associated with improved neurocognitive development and growth in infants, particularly in premature infants with low fat reserves [2]. Most commonly, breast milk is deficient in EPA and DHA, which are naturally sourced from fish, seafood, and seaweed [8]. A special relationship is noted between low maternal intake of DHA and its reduced concentration in breast milk. Low fish consumption among lactating mothers may be the reason for the deficiency of this acid in infants. DHA insufficiency may increase the risk of central nervous system dysfunction and impair the development of cognitive functions and visual processes [9].

Fat is one of the components of breast milk and is characterized by high variability. The quality of breast milk fat and its fatty acid composition is influenced by external factors such as the lactation period, the mother’s age, socioeconomic factors, body weight and height, and the woman’s established eating habits and lifestyle [10]. Breast milk is variable in composition during the first two months of life to adapt individually to the changing needs of the infant. Immediately after birth, colostrum is secreted, and its main function is in immunity [11]. Gila Diaz et al. [12] reported that colostrum contains a variety of bioactive compounds that stimulate the development and maturation of the infant’s immune system. After 7–10 days postpartum, the transitional milk is formed, which contains higher concentrations of energy and nutrients as the infant’s nutritional needs increase. It is reported that milk after 4–6 weeks postpartum is considered mature milk, and its composition remains unchanged until the end of lactation [6]. 

Infant formulas are alternative foods for infants and young children that are used when the mother is unable or chooses not to breastfeed [13,14]. The production of infant formula is very restrictive, and manufacturers must follow a series of standards to ensure that the composition of the final product covers all the nutritional needs of infants [15]. Over the past few decades, manufacturers have continuously improved the composition of infant formula to resemble breast milk to the highest possible degree [16]. Despite technological advances, large differences in the fatty acid composition of infant formula compared with breast milk, particularly of ALA, EPA, DHA, and AA, are still indicated [16]. Considering the high variability of the lipid fraction of breast milk, it is difficult to establish optimal target levels of fatty acids when developing infant mixtures [17].

The variability in the fatty acid profile of breast milk and infant formula has been a topic frequently discussed by researchers in recent times [18,19,20]. There are limited studies in the literature on the impact of lactation on the fatty acid profile among Polish women living in the Warmia and Mazury region. Accordingly, the aim of this study was to:

(1) Determine the differences between the fatty acid profile of colostrum, transitional milk, and mature milk of Polish women living in the Warmia and Mazury region;
(2) Compare the fatty acid profile of breast milk from different periods of lactation with that of commercially available infant formulas in Poland;
(3) Determine, using multivariate methods, which fatty acids are most responsible for the differences indicated between breast milk samples from different lactation periods and between breast milk and infant formulas.

## 2. Materials and Methods

### 2.1. Chemicals

Ammonia, potassium hydroxide, and sodium sulfate were obtained from Sigma Chemical Co. (St. Louis, MO, USA). Methyl and ethyl alcohol, diethyl ether, and petroleum ether were purchased from Merck (Darmstadt, Germany).

### 2.2. Milk Sample Collection

The study material involved maternal milk samples from three different stages of lactation (colostrum, transitional milk, mature milk) and two types of follow-on infant formulas. Samples of milk were obtained from 100 women living in north-eastern Poland in the Warmian–Masurian province. The opportunity to participate in the study was made available after meeting the following inclusion criteria: (1) good health; (2) breastfeeding the child and having no contraindications to natural feeding; (3) having undergone childbirth without complications. Breastfeeding women were required to fill out a questionnaire containing data on their age, place of residence, weight and height, past illnesses, and medications they have been taking. The second part of the questionnaire concerned data on pregnancy and breastfeeding (date and type of delivery, month of milk sample collection, date of milk sample collection). Women participating in the study gave written informed consent to participate. The characteristics of the study group of women are shown in Table S1.

Milk samples of 50–100 mL were collected from study participants using electric breast pumps or milk collectors immediately after breastfeeding. All mothers included in the study were informed in advance about proper milk storage (tightly closed tube, refrigerated conditions). When it was not possible to collect an adequate amount of milk during one feeding, women replenished the milk during the next feeding by refilling the same tubes. The milk samples prepared by the women were transported and stored in a Gorenje F6313W freezer (Velenje, Slovenia) at −30 °C in sterile tubes until analytical testing. Of the 100 study participants, 69 women provided sufficient amounts of milk. The study material gathered was categorized by lactation period as the criterion and included 21 colostrum samples, 26 transitional milk samples, and 22 mature milk samples. In addition, this study took into account two types of commercial follow-on infant formulas (for feeding infants older than 6 months) purchased in the Olsztyn market. Each infant formula was composed of cow’s milk and was in the form of a powder, which was mixed with water according to the instructions on the package. Table 1 summarizes the nutritional value and preparation of the infant formulas analyzed. The indicated information was obtained from the original packaging of the products. Table 2 shows the content of saturated, monounsaturated, and polyunsaturated fatty acids in 100 mL of ready-to-eat infant formula, expressed as % of total fatty acids. Calculations were made based on Table 1, using the information for the preparation of 100 mL of both kinds of milk.

### 2.3. Fat Extraction

Human milk samples were removed from the freezer, defrosted, and quickly mixed. They were then heated to 40 ± 1 °C for homogenization. The individual infant formulas were prepared according to the instructions on the package, as shown in Table 1 (the appropriate amount of powder was added to the correct amount of water). Fat from breast milk was extracted using the Rose-Gottlieb method [21]. For the weighed samples of human milk and infant formula (approximately 10 g was weighed to the nearest 0.01 g), 2 mL of 10% ammonia and 10 mL of ethanol were added, stirring gently each time. The fat was extracted from the prepared mixture with in 25 mL each of diethyl ether and petroleum ether. The upper organic layer was collected and then filtered through anhydrous sodium sulfate. Subsequently, using a rotary evaporator, the organic solvents were evaporated from the samples. Double extraction of milk fat was utilized.

### 2.4. Determination of the Fatty Acid Profile Using Gas Chromatography (GC)

The fatty acids of all types were converted into the appropriate fatty acid methyl esters (FAMEs) following the International Dairy Federation (IDF)’s standard method (2002) [21], utilizing a methanol solution of potassium hydroxide (KOH) [22]. The methyl esters obtained in the process were analyzed using the gas chromatography (GC) method. Chromatographic separation was carried out using a Hewlett-Packard 6890 gas chromatograph (Palo Alto, CA, USA) with a flame ionization detector (FID) and a Supelcowax 10 capillary column (length—100 m; inner diameter—0.25 mm; liquid phase—Supelcowax 30; film thickness—0.25 mm; temperature: detector = 250 °C, dispenser = 230 °C, column = 195 °C; carrier gas—helium; flow rate—1.5 mL/min) (Supelco, Sigma-Aldrich subsidiary, St. Louis, MO, USA). The fatty acid methyl esters were identified based on their retention times and were compared with those specified for the mixture of fatty acid methyl esters in a standard mixture of Supelco 37 Component FAME (10 mg/mL in methylene chloride). The G1701BA ChemStation B.01.00 software (Agilent, Alpharetta, GA, USA) was utilized to calculate the percentage of fatty acids. Individual fatty acids were expressed as a weight percentage of total FAMEs. 

### 2.5. Statistical Analysis

The data obtained were presented as the mean ± standard deviation (SD). The distributions of the studied variables in the samples were tested against a normal distribution using the Shapiro–Wilk test, and the homogeneity of variance was tested using Levene’s test. The tested distribution was not normal, and the variance was not homogeneous; therefore, non-parametric tests were used. The quantitative data collected between the distinguished groups (fatty acids in breast milk with regard to lactation and fatty acids in selected infant formulas) were compared using the non-parametric Kruskal–Wallis test. Principal component analysis (PCA) and cluster analysis (CA) were also carried out to show differences between breast milk samples according to the lactation period and to determine which fatty acids most determined these changes. The level of significance was assumed at *p* < 0.05. Statistical analysis was performed using Statistica 13.1 (Statsoft Inc., Tulsa, OH, USA).

## 3. Results 

### 3.1. Fatty Acid Profiles

The fatty acid composition is shown in Table 3. A total of 29 fatty acids were detected in HM and IF, including ten saturated fatty acids (SFAs), six monounsaturated fatty acids (MUFAs), five polyunsaturated fatty acids (PUFAs) (including three from the omega-3 family and two from the omega-6 family), three branched-chain fatty acids (BCFAs), and five trans fatty acids (TFAs).

#### 3.1.1. Saturated Fatty Acids (SFAs)

In both breast milk and infant formula, one acid from the short-chain fatty acid (SCFA) group (with 4–6 carbon atoms)—caproic acid (C6:0)—was detected. Its value was at the level of 0.03% and 0.01% in transitional and mature milk, respectively. In infant formulas, its content was significantly higher—0.21% and 0.17% in IF-I and IF-II, respectively (*p* < 0.05). 

Among medium-chain fatty acids (MCFAs), caprylic (C8:0), capric (C10:0), and lauric (C12:0) acids were identified. In breast milk, total MCFAs were 4.99%, 7.90%, and 8.48% in colostrum, transitional milk, and mature milk, respectively. In contrast, the MCFA content in infant formula was 16.97% in IF-I and 13.44% in IF-II, respectively. These values are on average about two to three times higher than in breast milk at different stages of lactation. 

Of the SFAs, the predominant acid in breast milk and infant formula was palmitic acid (C16:0), with contents of 28.10%, 29.24%, and 26.58%, respectively, in colostrum, transitional, and mature milk. In the present study, there was a decrease in C16:0 in mature milk relative to colostrum. The reported decline oscillated at around 6%. The percentage of C16:0 in infant formulas varied from one manufacturer to another. In IF-I the content was 18.82%, and in IF-II it was 21.68%. Compared with breast milk, the C16:0 content in infant formulas was about 30% lower. 

The second highest percentage of fatty acids in breast milk was myristic acid (C14:0) (5.94%—colostrum; 7.43%—transitional milk; 9.21%—mature milk), the amount of which increased significantly as the milk matured (*p* < 0.05). On average, infant formulas reported 30% and 40% lower C14:0 content relative to breast milk. The third dominant SFA in the milk studied was stearic acid (C18:0). There was a slight increase in its concentration as lactation progressed (6.72%, 7.13%, and 7.58% in colostrum, transitional milk, and mature milk). Infant formulas contained about two times lower C18:0 than human milk (*p* < 0.05). Of the other SFAs, pentadecylic acid (C15:0) (0.36–0.40% in HM and 0.04–0.06% in IFs), margaric acid (C17:0) (0.36–0.39% in HM and 0.07–0.09% in IFs) and arachidic acid (C20:0) (0.02–0.07% in HM and 0.29–0.33% in IFs) were present in lower amounts in the kinds of milk studied (*p* < 0.05).

#### 3.1.2. Monounsaturated Fatty Acids (MUFAs)

There were five MUFAs identified in breast milk and infant formula—myristoleic (C14:1 *n*-5), palmitoleic (C16:1 *n*-7), heptadecenoic (C17:1 *n*-9), oleic (C18:1 *n*-9), eicosenoic (C20:1 *n*-9) and ercucic (C22:1 *n*-9) acid. 

The C18:1 *n*-9 was the most prevalent, accounting for about 30% of the proportion of all fatty acids in both colostrum, transitional, and mature milk. For both types of infant formulas, the content of oleic acid was higher than in breast milk (*p* < 0.05) and was at about 37% and 35% of the proportion of total fatty acids in IF-I and IF-II, respectively. 

The content of erucic acid was dependent on the lactation period and decreased by 40% in mature milk relative to colostrum (*p* < 0.05). In contrast, the content of C14:1 *n*-5 was constant throughout lactation. A slight increase in C16:1 *n*-7 was observed in the study, the amount of which was 22% higher in transitional milk relative to colostrum and mature milk. In addition to higher C18:1 *n*-9 contents, the content of other MUFAs was significantly lower in both IFs than in human milk.

#### 3.1.3. Polyunsaturated Fatty Acids (PUFAs)

Among the fatty acids studied, there were differences in the PUFA content in different fractions of breast milk. The dominant *n*-3 PUFA in breast milk was ALA. Its content increased as lactation progressed: from 0.72% in colostrum to 0.94% in mature milk. An increasing trend was also noted for the most abundant acid in the PUFA *n*-6 group, LA—its content increased with as lactation progressed, from 8.49% in colostrum to 8.83% in mature milk. It should be noted that in the study conducted for both ALA and LA, there was an insignificant increase in their levels relative to the lactation period (*p* > 0.05). 

For other PUFA *n*-3 and *n*-6 acids, there was a significant decrease in DHA as lactation progressed. Relative to colostrum versus mature milk, its concentration decreased by 25%. In the case of AA, its level also decreased as lactation progressed, from 0.61% in colostrum to 0.47% in mature milk. EPA was stable regardless of the lactation period, and its level ranged from 0.15% to 0.18%. 

The PUFA profile in infant formulas differed from breast milk, especially in terms of the LA, ALA, and EPA content. The content of ALA in infant formulas differed from one producer to another. IF-I contained about 30% more ALA compared with IF-II. In both types of infant formula, the content of this acid was significantly higher than in breast milk (*p* < 0.05). A similar trend was noted for LA, with a higher amount observed in infant formulas (*p* < 0.05). Based on the study, it was observed that among omega-3 fatty acids, the level of EPA was significantly higher in breast milk (0.17% in colostrum, 0.18% in transitional milk, and 0.15% in mature milk) than in infant formula (0.03% and 0.04% in IF-I and IF-II, respectively). The two types of infant formulas contained similar DHA content, which was at the same level as the DHA in colostrum. On the other hand, both types of infant formula contained about 27–36% more DHA than mature milk (*p* < 0.05). In addition, infant formulas had similar levels of AA, with the same amount in transitional and mature milk. The AA content in both types of infant formulas was higher than the marked DHA levels.

#### 3.1.4. Branched-Chain Fatty Acids (BCFAs)

The study identified three fatty acids from the BCFA group—isopentadecylic acid (*iso* C15:0), anteisopentadecylic acid (*anteiso* C15:0), and isopalmitic acid (*iso* C16:0). The content of these acids was constant in breast milk, and the predominant acid was *anteiso* C15:0 (0.10–0.12%).

While HM contained the highest amounts of *anteiso* C15:0, IF-II contained only trace amounts of this acid. The second most abundant BCFA in HM was *iso* C16:0 (0.07–0.09%), which was found in 0.06% in IF-I. Both IFs did not contain *iso* C15:0 in their composition. 

#### 3.1.5. Trans Fatty Acids (TFAs)

There were five types of trans fatty acids identified in milk samples—petroselaidic and elaidic (C18:1 *n*6+*n*9 *t*) acid; vaccenic acid (C18:1 *t*11, VA); conjugated linoleic acid (CLA), a *trans* isomer of linoleic acid; rumenic acid (C18:2 *c*9*t*11, RA); and palmitelaidic acid (C16:1 *t*7). 

The predominant TFA in breast milk was VA, with levels of 2.99%, 1.74%, and 1.52% in colostrum, transitional milk, and mature milk, respectively. Each fraction of breast milk contained RA, but the values varied depending on the lactation period. The average RA content was 0.21% in colostrum, and 0.13% and 0.18% in transitional and mature milk, respectively. The level of RA was shown to be significantly lower in transitional milk (*p* < 0.05). Of the other TFAs, petroselaidic and elaidic acid were identified, which totaled 0.56% in colostrum, 0.45% in transitional milk, and 0.49% in mature milk. In the study conducted, the lowest amounts of palmitelaidic acid were identified in the tested milk samples. 

Significantly lower contents of selected TFAs were identified in infant formulas. The detected TFAs were petroselaidic and elaidic acid, accounting for 0.08% and 0.15% of all fatty acids in IF-I and IF-II, respectively. Infant formulas did not contain any other acids from the TFA group. 

### 3.2. The Average Content of SFAs, MUFAs, PUFAs, BCFAs, and TFAs, and the Relationship between Selected Fatty Acids

#### 3.2.1. Human Milk 

Both colostrum, transitional milk, and mature milk were richest in SFAs (46.50%; 52.54%; 52.71%, respectively) and MUFAs (34.24%; 33.27%; 31.88%, respectively), followed by PUFAs (10.43%; 10.54%; 10.72%, respectively). The total SFA concentration increased from 46.50% in colostrum to 52.71% in mature milk. MUFA concentrations remained stable in colostrum and transitional milk (34.24% and 33.27%, respectively), while they decreased in mature milk to 31.88%. (*p* < 0.05). On the other hand, there was an increase in PUFA *n*-3 from 1.33% in colostrum to 1.42% in mature milk, although it was not a significant growth. A downward trend in the PUFA *n*-6:*n*-3 ratio was observed during lactation. The highest ratio was observed in colostrum (6:84:1), followed by a significant decrease in transitional and mature milk (6.37:1 and 6:55:1, respectively). In the case of TFAs, there was a significant decrease in these acids as lactation progressed. While colostrum contained 3.86% TFAs, transitional and mature milk contained about 40% less of these acids. 

In the present study, the ratio of PUFA *n*-3 acids or PUFA *n*-3 acids to PUFA *n*-6 acids was evaluated. The relationship between DHA and LA was 0.05:1 in colostrum and transitional milk and 0.04:1 in mature milk. Another important post-experimental function is the balance between DHA and AA. In the studies conducted, it ranged from 0.70:1 in mature milk to 0.79:1 in transitional milk. The third fatty acid ratio assessed was the ratio between LA and ALA. In the study, the value decreased as the milk matured, from 11.79:1 in colostrum to 9.39:1 in mature milk. 

#### 3.2.2. Infant Formulas 

Infant formulas contained the same acid proportions as human milk - the highest percentage of SFA and the lowest PUFA. IF-I contained significantly more MUFAs compared with breast milk from different lactation periods (*p* < 0.05). Significant differences were found between PUFA percentages. IF-I samples contained almost 20% less PUFAs, including more than 20% less *n*-6 PUFA, compared to IF-II. The TFA content was 0.08% in IF-I and 0.15% in IF-II.

Based on information from the labels of infant formulas, the percentage of fatty acid groups was calculated: SFAs, MUFAs, and PUFAs in each of the formulas (Table 2). In the study conducted, the sum of SFAs was 45.01% and 44.06% in IF-I and IF-II, respectively. Based on the calculated values, the manufacturer declared the presence of SFAs at 43.65% and 44.60% in IF-I and IF-II, respectively. In the studies conducted, the content of BCFAs (classified as SFAs) was reported separately, and when the labeled SFAs and BCFAs were added together, values of 45.07% and 44.07% in IF-I and IF-II were obtained. In the case of MUFAs, the identified values in IF-I and IF-II were 38.03% and 35.47%, respectively, and 38.11% and 35.62% after taking into account selected TFAs (C18:1 *n*6+*n*9 *t*) counted as MUFAs. The manufacturer declared MUFA levels of 39.68% and 39.57%, which were higher than those determined by the authors by about 1% and 4%, respectively. The last group of fatty acids—PUFAs—was present in IF-I and IF-II in the amount of 15.84% and 19.15%, while the values indicated by the manufacturer were 16.67% and 15.83%. In this case, the marked values were lower than those declared by the manufacturer by 1% in IF-I and exceeded the values by more than 3% in IF-II. The ratio of LA:ALA in infant formulas ranged from 6:05:1 for IF-I to 10.72:1 in IF-II. On the other hand, the ratio of DHA:AA acids was 0.81:1 and 0.92 in IF-I and IF-II, respectively. In contrast, the ratio of DHA:LA acids in infant formula was 0.03:1 in both IF-I and IF-II. 

### 3.3. Associations between Obtained Data—Multivariate Analysis

#### 3.3.1. Principal Component Analysis (PCA)

**Figure 1:**
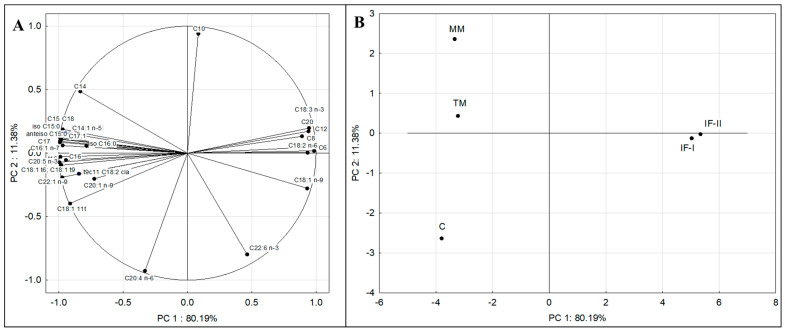
The principal component plot showing variations in the selected fatty acids (C6:0, C8:0, C10:0, C12:0, C14:0, C15:0, C16:0, C17:0, C18:0, C20:0, C14:1 *n*-5, C16:1 *n*-7, C17:1 *n*-9, C18:1 *n*-9, C20:1 *n*-9, C22:1 *n*-9, C18:3 *n*-3, C20:5 *n*-3, C22:6 *n*-3, C18:2 *n*-6, C20:4 *n*-6, *iso* C15:0, *anteiso* C15:0, *iso* C16:0, C18:1 *n*6+*n*9 *t*, C18:1 *t*11, C18:2 *c*9*t*11, C16:1 *t*7) of the analyzed infant formulas and human milk samples (**A**) and the score plot of the analyzed human milk samples from different lactation periods and selected infant formulas (**B**). Explanations: C—colostrum, TM—transitional milk, MM—mature milk; IF-I, IF-II—infant formulas from different manufacturers.

Principal component analysis (PCA) was used to explain the structure of variation in the collected data on the fatty acid profile of breast milk from different periods of lactation (colostrum, transitional milk, mature milk) and to compare it with the structure of variation in infant formulas. The PCA was conducted among all the breast milk samples tested, and the variables included various fatty acids (C6:0, C8:0, C10:0, C12:0, C14:0, C15:0, C16:0, C17:0, C18:0, C20:0, C14:1 *n*-5, C16:1 *n*-7, C17:1 *n*-9, C18:1 *n*-9, C20:1 *n*-9, C22:1 *n*-9, C18:2 *n*-6, C20:4 *n*-6, C18:3 *n*-3, C20:5 *n*-3, C22:6 *n*-3, *iso* C15:0, *anteiso* C15:0, *iso* C16:0, C18:1 *n*6+*n*9 *t*, C18:1 *t*11, C18:2 *c*9*t*11, C16:1 *t*7). Two principal components described 91.57% of the variance. Figure 1A presents the relationships between individual fatty acids and the resulting principal components. All variables, except C10:0, C20:4 *n*-6, and C22:6 *n*-3, were transferred to a greater extent by PC1. Based on the PCA analysis, a positive correlation was found between selected SFAs (C6:0, C8:0, C12:0, C20:0) and acids from the MUFA and PUFA groups (C18:1 *n*-9, C18:2 *n*-6, C20:4 *n*-6, C18:3 *n*-3, C22:6 *n*-6). Strongly positively correlated acids were found between the BCFA and TFA groups. On the other hand, there was a strongly negative correlation between PUFAs (C18:2 *n*-6, C18:3 *n*-3) and selected MUFAs (C14:1 *n*-5, C16:1 *n*-7, C20:1 *n*-9), SFAs (C14:0, C16:0, C17:0, C18:0), and TFAs (C18:1 *n*6+*n*9 *t*, C18:1 *t*11, C18:2 *c*9*t*11, C16:1 *t*7).

Figure 1B shows a plot of points in the principal component plane that shows the similarities and differences between the fatty acid profiles of human milk from three lactation periods and two types of follow-on infant formulas. The arrangement of the analyzed cases around each other indicates that human milk from different lactation periods had different fatty acid profiles and differed from the profiles of selected infant formulas. The figure shows that among the breast milk fractions, the transitional milk (TM) and mature milk (MM) samples are the most similar in terms of fatty acid content. It was observed that C significantly differs from TM and MM on the graph, which indicates a particular variability in the fatty acid profile between colostrum and transitional and mature milk. Infant formulas (IF-I and IF-II) were on the opposite side of the graph, which indicates their different fatty acid profiles concerning human milk from each lactation period. The graph shows that of the breast milk fractions, the transition milk (TM) and mature milk (MM) samples are the most similar in terms of fatty acid content. 

The distribution of cases in Figure 1A can be attributed to the different fatty acid profiles of individual infant milk samples. The distribution of case C, clearly separated from TM and MM, is the result of increased TFA content, mainly of VA and also AA. Based on the graph, the separation of TM and MM was the effect of the presence of higher amounts of C14:0, C18:0, *iso* C16:0, and C14:1 *n*-5 in them. In the case of C14:0, the difference in content was significant (for mature milk), but the content of the other acids mentioned above did not differ significantly. In the case of infant formulas, IF-I was differentiated mainly in terms of C18:1 *n*-9, while for IF-II, it was by C18:2 *n*-6. The differences in composition between infant formula and human milk explain the much higher amounts of TFAs and BCFAs contained in human milk.

#### 3.3.2. Cluster Analysis (CA)

**Figure 2:**
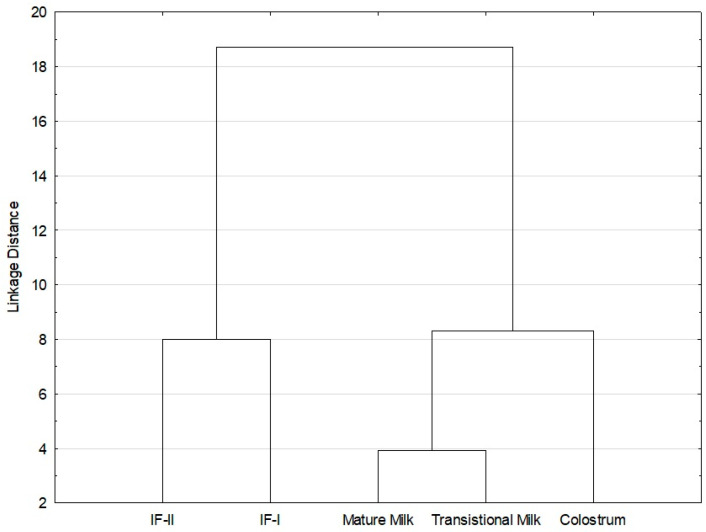
Cluster analysis (CA) of the fatty acid profile in studied human milk from different lactation periods and infant formulas. Abbreviations: IF-I, IF-II—infant formulas from different manufacturers.

Cluster analysis (CA) was also used to assess the variation in fatty acid profiles in human milk from different lactation periods and selected infant formulas. Based on the content of individual fatty acids (C6:0, C8:0, C10:0, C12:0, C14:0, C15:0, C16:0, C17:0, C18:0, C20:0, C14:1 *n*-5, C16:1 *n*-7, C17:1 *n*-9, C18:1 *n*-9, C20:1 *n*-9, C22:1 *n*-9, C18:2 *n*-6, C20:4 *n*-6, C18:3 *n*-3, C20:5 *n*-3, C22:6 *n*-3, *iso* C15:0, *anteiso* C15:0, *iso* 16:0, C18:1 *n*6+*n*9 *t*, C18:1 *t*11, C18:2 *c*9*t*11, C16:1 *t*7), a cluster dendrogram was constructed. It was determined by the weighted pair–group average method, and distances were measured using the Euclidean distance formula method. In the CA analysis, similar objects are combined into clusters. The vertical scale of the dendrogram shows the distance or dissimilarity; small values indicate that they are in the same cluster. The dendrogram in Figure 2 shows one main cluster divided into smaller clusters. One subgroup contains all the fractions of human milk and the other, the infant formulas. The results of the cluster analysis confirmed the results of the principal component analysis. The figure indicates that the most similar in terms of fatty acid composition are the mature milk and transitional milk samples and, to a lesser extent, they are similar to colostrum. The dendrogram indicates different fatty acid profiles between human milk and infant formulas. The colostrum samples were most diverse relative to IF-I and IF-II.

## 4. Discussion

### 4.1. Fatty Acid Profiles

Breast milk undergoes dynamic compositional changes in terms of selected external factors [3]. The study examined changes in the fatty acid profile of human milk samples of women from the Warmia and Mazury region with regard to lactation periods. The results obtained were compared with two types of follow-on feeding infant formulas to show the similarities and differences between the two study materials. Based on the study, there is a significant impact of the lactation period on the quantitative changes in selected fatty acids over time.

#### 4.1.1. Saturated Fatty Acids (SFAs)

Short-chain fatty acids (SCFAs) include acids containing one to six carbon atoms. This group of acids includes the following: formic (C1:0), acetic (C2:0), propionic (C3:0), butyric (C4:0), valeric (C5:0), and caproic (C6:0) acids. They play an important role in the infant’s body: first of all, they are substrates of glucose, cholesterol, and lipid metabolism; in addition, they are responsible for maintaining the metabolic balance and controlling inflammation [23]. Khor et al. [6] detected significantly higher amounts of C6:0—0.11% and 0.13% in transitional and mature milk—than the authors in this study (0.03% and 0.01%). Infant formulas contained significantly higher amounts of C6:0, which was attributed to the higher amount of this acid in cow’s milk, on which the infant formulas were based, than in breast milk (1% vs. 0.1%) [24].

In the study, total MCFAs (C8:0, C10:0, and C12:0) increased from 4.99% in colostrum to 8.48% in mature milk. In the study by Floris et al. [2], these acids were 4.06%, 7.61%, and 8% in colostrum, transitional milk, and mature milk, confirming the trend of increasing MCFAs in breast milk as lactation progresses. In the case of C10:0 and C12:0, similar conclusions were reached by Giuffrida et al. [19]. The MCFA content in infant formulas was significantly higher than in breast milk (16.97% in IF-I and 13.44% in IF-II), and this difference is mainly explained by the different fat sources used in infant formula production, including the addition of coconut and palm oils, which contain high amounts of MCFAs [25]. MCFAs provide an essential source of energy for infants. Unlike long-chain fatty acids (LCFAs), they are absorbed and oxidized more easily to produce energy. Their addition to infant formulas may promote better lipid absorption [26]. In addition, these acids have antiviral, antimicrobial, and modulatory effects on the intestinal microflora, especially in early infancy [26]. Infant formulas contained significantly higher contents of SCFAs (0.21% and 0.17% in IF-I and IF-II) and MCFAs (16.97% and 13.44% in IF-I and IF-II) relative to breast milk (*p* < 0.05). The acids in these groups are commonly included in infant formulas as they can be directly absorbed through the portal vein and are a fast source of energy for infants [27]. In the case of lauric and myristic acids, it is recommended that their total content in infant formulas should not exceed 20% of total fatty acids; in this example, amounting to 18.33% and 14.73% in IF-I and IF-II, respectively. It is reported that too high a supply of these acids raises the plasma levels of total cholesterol and low-density lipoprotein (LDL), which is associated with an increased risk of cardiovascular disease [17]. 

Palmitic acid predominated in the human milk and infant formulas studied. The C16:0 content decreased slightly as lactation progressed, similar to the study by Floris et al. [2]. It is reported that the C16:0 content of human milk is constant and independent of the lactation period [2]. In contrast, the C16:0 content in infant formulas was lower than in human milk. In the Wu et al. [28] study, one of the infant formulas contained 29.21% of this acid, while the other contained 10.08%. In this case, it was justified by the addition of 1,3-dioleoyl-2-palmitoylglycerol (OPO), which contains significant amounts of C16:0. In the case of C16:0 acid in infant formulas, it is important to consider the form in which the acid occurs. In breast milk, palmitic acid is found in the *sn*-2 position [18,29]. On the other hand, C16:0 is mainly found in infant formulas in the *sn*-1 or *sn*-3 position. It has been indicated that C16:0 acid in the *sn*-2 position promotes better absorption of calcium and fat and reduces the risk of skeletal problems in breast-fed infants [18,29]. For this reason, triglycerides in infant formula are increasingly being modified and enriched with palmitic acid in the *sn*-2 position, which enables better calcium and fat absorption in newborns [30].

Changes in C14:0 content were observed relative to maturing milk, with the content increasing from 5.94% in colostrum to 9.21% in mature milk. The same trend was noted by Bousset-Alféres et al. [30] and Giuffrida et al. [19]. In the case of stearic acid, only a slight, insignificant increase in its amount relative to the duration of lactation was observed, as in the Floris et al. [2] and Wu et al. [28] studies. Giuffrida et al. [19] report that C18:0 levels are non-variable throughout lactation. Concerning C18:0 in infant formulas, the values obtained in these studies were lower than those reported in the literature [16,28].

#### 4.1.2. Monounsaturated Fatty Acids (MUFAs)

The content of C18:1 *n*-9 in breast milk comprised more than 90% MUFAs, which is in line with studies presented by other authors [20,31]. A slightly higher (35.56%) oleic acid content than in the presented study was shown by Wu et al. [28]. In a study by Sánchez-Hernández et al. [17], C18:1 *n*-9 levels were 34%, 32%, and 50% higher in colostrum, transitional, and mature milk, respectively. This may be explained by the practice of the Mediterranean diet in Spain and the widespread consumption of olive oil. The C18:1 *n*-9 content is reported to be stable throughout lactation. In the present study, there were no significant differences in the content of this acid relative to the lactation period, similar to that found by Floris et al. [2]. In addition to providing energy reserves, oleic acid controls the synthesis of medium-chain fatty acids and serves important transport and metabolic functions [17]. In infant formulas, the C18:1 *n*-9 content was higher, which may be explained by the high proportion of vegetable oils (canola, sunflower, palm, and coconut) used in their production, which are excellent sources of C18:1 *n*-9 acid [28]. 

The content of other MUFAs in breast milk was 2.70–3.22%, while in infant formulas, it was 0.37–0.69%. Most MUFAs remain stable during lactation; however, the levels of some acids (eisosenoic, ercucic) may decrease as the milk matures [2]. Such a trend in the study was noted for ercucic acid, whose content decreased by 40%. A decreasing trend for changes in the content of this acid was also observed in the studies by Bousset-Alféres et al. [30] (1.78%—colostrum, 0.82%—transitional milk, 0.76%—mature milk) and Moltó-Puigmarti [32] (0.27% colostrum, 0.12%—transitional milk, 0.10%—mature milk). The content of C14:1 *n*-5 was constant throughout lactation, as in the Sánchez-Hernández et al. [17] study. In the studies conducted, there was an increase in C16:1 *n*-7 acid, the amount of which was 22% higher in transitional milk relative to colostrum and mature milk. A similar trend was noted by Khor et al. [6]. In addition to higher C18:1 *n*-9 contents, the content of other MUFAs was significantly lower in both IFs than in human milk. It has been reported that the content of MUFAs having more than 18 carbon atoms (ercucic, eicosenoic, nervonic acids) decreases as lactation progresses. MUFAs with shorter chains can be endogenously converted into longer-chain acids. As the infant’s metabolic pathways in the first days of life may not be completely formed and functional, it is important to provide colostrum to the infant to supply MUFAs with longer chains [32]. It should be noted that these fatty acids have a very important metabolic function, taking part in the biosynthesis of myelin and stimulating the development of the infant’s nervous system [30].

#### 4.1.3. Polyunsaturated Fatty Acids (PUFAs)

##### Human Milk

For PUFAs, there was a significant effect of the lactation period on the levels of selected acids, as reported by Sanchez Hernandez et al. [17] and Zou et al. [33]. The content of ALA slightly increased as lactation progressed, similar to studies by Nilsson et al. [34] and Giuffrida et al. [19]. The detected level of this acid was more than twice as high as in the study by Khor et al. [6]. In contrast, Giuffrida et al. [19] detected the same amounts of ALA in mature milk as in our study. As lactation progressed, an increase in the most abundant acid in the PUFA group—LA—was observed. However, relative to the study by Sanchez-Hernandez et al. [17], who observed a 3% increase in LA relative to colostrum vs. mature milk, in our study, this increase was only about 0.34%. Bousset-Alféres et al. [30] noted a similar increasing trend in LA as lactation progressed, although its content was more than 50% higher in each fraction of breast milk. LA and ALA are classified as essential fatty acids (EFAs) because, in contrast to other fatty acids, the human body is unable to produce them, and they must be supplied to the body from the diet. These acids play a significant role in the development of the young body. According to an EFSA report [35], infants consuming inadequate amounts of LA from birth had clinical evidence of deficiency of this acid, manifested in growth deficiencies and dermatological problems, among others. When adequate amounts of ALA are not provided, deficiency symptoms mainly include neurological and behavioral problems. 

Adequate PUFA levels in breast milk significantly affect fetal development, primarily on nervous system formation [36]. During the first six months of life, the most dynamic development occurs in the child’s nervous system. The intensity of metabolic processes increases to such a degree that meeting the baby’s needs is a major challenge for women. An adequate supply of LA, ALA, DHA, and AA is necessary during this time [37]. Linoleic acid is a precursor of arachidonic acid, while α-linolenic acid is a precursor of docosahexaenoic acid; therefore, their presence is extremely important in the synthesis of long-chain polyunsaturated fatty acids (LCPUFAs). In general, our study reported slightly lower values of α-linolenic acid (about 0.7–0.9%) and linoleic acid (about 8–9%) compared with other research results (1.0–1.40%—ALA; 8–11%—LA) [6,34,38]. It should be noted that the PUFA content of breast milk is largely determined by external factors, including the diet of the nursing woman [36]. According to several available studies, a correlation is indicated between the LA and ALA content in human milk and the consumption of vegetable oils and marine fish [6,39]. Isesele et al. [40] report that obese women have higher levels of LA in milk than women with a normal BMI. LA, as a precursor of AA, is converted to it through enzymatic reactions (involving delta-6-desaturase, elongase, and delta-5-desaturase). Obesity correlates with increased delta-6-desaturase activity; therefore, obese women may have higher levels of AA in breast milk in addition to higher levels of LA.

LCPUFAs (AA, EPA, and DHA) represent a small percentage of the composition of breast milk; however, they have a significant impact on the development of the newborn. They promote the development of the nervous system, stimulate cognitive function, and ensure normal somatic development [5]. DHA content decreased with lactation, with higher content in colostrum and decreasing levels in transitional and mature milk. A decreasing trend for DHA was also noted by Nilsson et al. [34], who observed a 50% decrease in this acid with the progression of lactation, while Bousset-Alféres et al. [30] noted a 66% decrease. Other conclusions were reached by Khor et al. [6], who indicated a slight increase in DHA relative to lactation, with its highest level in mature milk. The global level of DHA in breast milk is 0.37 ± 0.11%, and its level can vary from 0.18% to as much as 1% of all fatty acids. DHA content in breast milk is linearly correlated with the proportion of this acid in the diet of nursing mothers. A higher intake of fish and seafood will result in higher levels of DHA in milk [41]. It is crucial to consume adequate amounts of fish, seafood, and fish oil, and to use an appropriate supplementation dose (200 mg of DHA daily; if fish consumption is low, higher supplementation, such as 400–600 mg, should be considered) [42]. Koletzko [43] states that “*breastfeeding women need to achieve a daily DHA intake of at least 200 mg to provide milk with a DHA content of at least 0.3%, which is required for a fully breastfed infant to achieve a daily supply of about 100 mg DHA / day considered desirable to meet metabolic needs*”. In the context of the fatty acid profile in breast milk, in addition to the dietary aspect, an important factor is the BMI. Hua et al. [44] showed that women with an increased BMI (overweight or obese) had lower proportions of PUFAs in their milk, including ALA, EPA, DHA, and higher ratios of AA/EPA and *n*6-*n*3. The effect of BMI on the fatty acid profile of breast milk is closely related to diet, as a higher intake of highly processed foods may modify fatty acid metabolism, thereby resulting in lower levels of circulating *n*-3 fatty acids and higher levels of *n*-6 fatty acids [45].

Colostrum similarly contained higher levels of AA in contrast to transitional and mature milk. The same trend was observed by Sanchez-Hernandez et al. [17] and Giuffrida et al. [19]. However, the AA content in our study was lower than that identified by Moltó-Puigmartí et al. [32] (0.61% vs. 0.92%—colostrum; 0.52% vs. 0.62%—transitional milk; 0.47% vs. 0.49%—mature milk). Regardless of the level, both studies showed a similar trend of decreasing AA content as the milk matured. It should be noted that the average AA content in the milk samples tested, 0.53%, was close to the global level of 0.46% [46]. 

LCPUFAs are important for many cellular processes, including normal growth, regulation of inflammation, and neurological development [18]. DHA performs important functions in the proper maturation of a baby’s nervous system; hence, adequate amounts of DHA are essential for the young body during the initial feedings [6]. AA also participates in the formation of the infant’s nervous system and is closely linked to intelligence and the formation of visual processes [47]. As was reported previously, AA and DHA can be synthesized from LA and ALA, however, these mechanisms are limited in infants. As infants grow and mature, their ability to synthesize AA and DHA improves, and thus the levels of such acids in milk gradually decrease [48]. Therefore, higher levels of AA and DHA in colostrum may be associated with benefits to the newborn and the delivery of adequate levels of these acids. In the presented study, the higher levels of AA and DHA in colostrum relative to mature milk can be justified by the need for a higher intake of these acids by newborns due to their reduced synthesis from LA and ALA in the first moments of life.

##### Infant Formulas

Over the past few decades, numerous studies have been conducted to make infant formulas as similar as possible to breast milk, especially in terms of the content of selected PUFAs—linoleic and α-linolenic acids [6]. The content of LA and ALA in infant formulas was significantly higher than in human milk (14.69% vs. 8.64% —LA; 1.83% vs. 0.83%—ALA). According to the guidelines, linoleic acid in infant formulas should be present in an amount of 7–20% of the fatty acid proportion. This amount of LA is adequate to cover the need for this ingredient, while a higher content may be associated with effects that influence lipoprotein metabolism, immune function, and levels of oxidative stress [16]. In the case of ALA, its level should not exceed 2% in IFs [15]. It is reported that the level of this acid is higher than in breast milk. This is mainly related to regulations on composing infant formulas in terms of PUFA content. The level of ALA in human milk can fluctuate depending on the mother’s race, her origin, and the proportion of individual sources of fat in the diet, so it is difficult to establish reference values for this acid [17,49].

Essential fatty acids (EFAs) are among the compounds that the body is unable to synthesize on its own due to the lack of selected enzymes (desaturase and hydrogenase) [16]. There were significant changes observed in the amount of LCPUFAs in infant formulas relative to human milk for both EPA, DHA, and AA. While EPA levels were lower in both types of infant formulas, DHA and AA levels varied relative to human milk depending on the selected lactation periods. The DHA content in both types of infant formulas was similar to colostrum and transitional milk, although it differed from the level of this acid in mature milk. In addition, the level of DHA in both types of IFs was similar, based on regulations determining the addition of DHA to IFs. According to a European Union regulation, from February 2020, manufacturers of infant formulas are required to contain DHA in milk in an amount of 20–50 mg/100 kcal (about 0.33–1% of total fatty acids) [50,51]. The infant formulas tested met the standards for DHA acid content. It is reported that these values are often higher than the average content of DHA in breast milk, and such results were also obtained in other conducted studies [52]. It should be noted that DHA is not directly synthesized in the human body; therefore, the infant must receive adequate amounts of it in breast milk [53]. However, AA and DHA can be produced endogenously with LA and ALA acids. On the other hand, this synthesis in newborns is limited due to the immaturity of the body and enzymatic processes. Therefore, newborns need to be supplied with AA and DHA immediately after birth. For breastfeeding women, giving colostrum to the newborn will fully meet the need for DHA [3]. Mandatory enrichment of infant formulas with DHA ensures that non-breastfed infants will also be provided with adequate amounts of this acid.

AA levels were similar in both types of infant formulas. According to European standards, the level of AA must not exceed 1% of total fatty acids [54]. Since 2020, manufacturers have been obliged to add DHA in strictly defined amounts to infant formulas; however, the regulation does not provide requirements for the amount of AA [55]. The Codex Alimentarius revision on the 2023 aspect of the Standard for Follow-Up Formula for Older Infants and Products for Young Children [56] includes recommendations for the addition of arachidonic acid to infant formulas. According to the regulation, if the follow-on infant formula contains DHA, the level of AA should be at least in the same amount as the amount of DHA; such a relationship was noted in the presented study. According to Koletzko et al. [55], providing high doses of DHA without sustainable amounts of AA can negatively affect infants in terms of non-optimal neurodevelopment and development of the immune system.

#### 4.1.4. Branched-Chain Fatty Acids (BCFAs)

Branched-chain fatty acids (BCFAs) are among the SFAs that have one or more methyl branches in the carbon chain. These acids are designated as *iso* or *anteiso*, depending on the presence of an isopropyl or isobutyl group [57]. In the study conducted, the predominant acid was *anteiso* C15:0, with a level of about 0.11% of the proportion of all fatty acids. Its content was significantly lower than in the study of Dingess et al. [57]. The authors identified the content of this acid at 0.56–1.91%, depending on the origin of the women. The content of BCFAs in breast milk significantly depends on the diet, especially on the consumption of dairy products and beef [58]. Dingess et al. [57] report that in a study conducted, women from Cincinnati (USA) consumed significantly more dairy products, which translated into higher levels of *anteiso* C15:0 (1.91%) in breast milk compared with women from Mexico (1.34%) and Shanghai (0.56%). A major limitation of this study is the lack of information on the current and habitual food intake of lactating women. However, according to statistics, milk consumption in Poland is much lower than in the United States (US), and there is still a decreasing trend in their consumption [59]. 

Infant formulas contained only trace amounts of BCFAs. Currently, manufacturers do not undertake to add BCFAs to infant formulas due to the complicated and time-consuming procedure of obtaining these acids from native raw materials. Wang et al. [60] attempted to enrich infant formulas with selected BCFAs from lanolin. The authors’ results indicated that the prepared concentrate had a similar composition and type of BCFAs to human milk fat and could be used to enrich infant formulas. It is reported that BCFAs, both *iso* and *anteiso* forms, have documented anticancer activity, and their anticancer potential may be stronger even than conjugated linoleic acid (CLA) [60]. In addition, Lucas and Cole [61] and Mcguire and Anthony [62] indicated that infants fed breast milk were less likely to be at risk for necrotizing enterocolitis (NEC). In contrast, Ran-Ressler et al. [63] reported that BCFAs can significantly reduce the incidence of NEC. Therefore, the addition of BCFAs to infant formulas could provide health benefits for infants, especially for babies born prematurely and having a low birth weight [60].

#### 4.1.5. Trans Fatty Acids (TFAs)

The content of TFAs is largely determined by the diet of lactating women. Individual TFAs can derive from two sources: natural (via biohydrogenation in the rumen), found in meat, milk, and milk products (vaccenic acid, rumenic acid), and from industrial production (via frying, grilling, hydrogenation of oils) such as in confectionery, bakery products, and French fries (elaidic acid, linoelaidic acid) [64].

Maternal milk was dominated by VA, whose content decreased from 2.99% in colostrum to 1.52% in mature milk. De Souza Santos da Costa et al. [65] indicate much lower VA values—0.76% in colostrum and 0.67% in mature milk. Similarly, Martysiak-Żurowska [66] presents a much lower range of VA content in breast milk (0.34–1.03%). The VA content of breast milk is largely determined by diet, especially the consumption of products rich in meat and meat products [64]. It can be assumed that the higher VA content in the studied group of women is a result of its higher intake. In addition, the observed decline in VA as lactation progresses may also be explained by changes in the diets of lactating women; in this example, by the reduced consumption of meat and its products. 

Conjugated linoleic acids (CLAs) are an intermediate product of the hydrogenation of PUFAs—LA and ALA—in the digestive tract of ruminants [66]. The most common CLA is rumenic acid (RA) (C18:2 *cis*-9-*trans*-11), which represents more than 93% of the total CLAs present in ruminant milk [67]. CLA is one of the few animal-derived compounds that exhibits potent biological activity and anticancer properties [68]. In this study, RA concentrations in colostrum and mature milk were the same; however, they decreased in the transitional milk fraction. It is reported that the presence of CLAs in the human organism is associated with the consumption of selected food products [66]. Martysiak-Żurowska et al. [66] reported that among lactating women on a standard diet without limiting milk and dairy products, RA levels ranged from 0.32–0.57%, while they were 0.14–0.33% among women on a dairy-poor diet. In the study conducted, the lack of applied information on the diets of the lactating women studied prevents a proper interpretation of the RA content in breast milk. Of the TFAs that are formed during the industrial hydrogenation of vegetable oils, two acids were identified—petroselaidic and elaidic acid—which were expressed as a sum. Their content remained constant in breast milk and averaged 0.5%. Bousset-Alféres et al. [30] found elaidic acid levels ranging from 1.334% in colostrum and 0.585% in mature milk; however, petroselaidic acid was not detected. The lowest amounts of palmitelaidic acid were detected in the milk of the women studied. In a study by De Souza Santos da Costa et al. [65], trans isomers of C16:1 acid also accounted for the lowest percentage in tested milk samples.

Infant formulas were poor in TFAs compared with breast milk. Of the five TFAs abundant in maternal milk, IFs contained only small amounts of petroselaidic and elaidic acids (0.08% and 0.15%). This is related to the fact that the fat in infant formulas comes exclusively from vegetable oils (palm, canola, coconut, sunflower). Martysiak-Żurowska [66] identified trace amounts of RA and VA in infant formulas. The presence of small amounts of RA and VA is related to the presence of nonfat cows’ milk in infant formulas, which may contain trace amounts of these compounds [51].

### 4.2. The Average Content of SFAs, MUFAs, PUFAs, BCFAs, and TFAs, and the Relationship between Selected Fatty Acids

#### 4.2.1. Human Milk

The results of each fatty acid group can be ordered as SFA > MUFA > PUFA, similar to the study by Bahreynian et al. [69]. In contrast, Wu et al. [28] indicated that breast milk was the richest in MUFAs, while Zhang et al. [70] pointed to PUFAs as comprising the highest percentage. These differences can be attributed to several factors, which primarily include individual variability, maternal dietary habits, geographic region, cultural differences, or socioeconomic factors [69]. Similar to the study by Khor et al. [6], our study observed an increase in SFA content in colostrum relative to mature milk. SFA content in the milk of lactating women worldwide ranges from 35.0–54.5% [70]. The average SFA content in the milk of Polish women is 43.6%, while it is much lower among residents of Greece (35.5%), Turkey (30.8%), or African countries (Nigeria—27.5%, Israel—33.1%, Democratic Republic of Congo (28.7%). Mediterranean countries (Greece, Turkey) popularize the Mediterranean diet, in which the consumption of red meat is reduced in favor of seafood, while countries such as Poland, Germany, and Hungary are among those where the consumption of meat and its products is relatively high [10]. There are indications of possible correlations between the consumption of cholesterol-rich products and SFA levels in human milk [71,72]. Di Maso et al. [73] report that adherence to Mediterranean dietary recommendations, including the consumption of high amounts of fish and seafood and reducing the proportion of fatty dairy products in the diet, are associated with lower levels of SFAs, especially C16:0 and C18:0, in breast milk. Another study found a weak positive correlation between the consumption of high-fat dairy products and SFA levels in maternal milk [74]. 

The MUFA content was constant in colostrum and transitional milk; however, it decreased significantly in mature milk, as in the study by Khor et al. [6]. This is related, on the one hand, to decreasing levels of MUFAs containing more than 18 carbon atoms (ercucic, eicosenoic). On the other hand, the type of diet consumed may also affect total MUFA levels in human milk, although to a significantly lesser extent than for PUFAs [75]. There are associations between olive oil and seafood consumption and MUFA levels in breast milk [76,77]. On the other hand, PUFA *n*-3 content increased as lactation progressed. Changes in the content of ALA and DHA can be explained primarily by women’s dietary habits. In countries where the Mediterranean diet is popularized (Spain, Croatia, Turkey), based on products containing significant amounts of omega-3 fatty acids (fish, seafood), the levels of PUFA in breast milk are higher (18.4–26.9%) than in Central and Eastern European and Scandinavian countries (11.9–15.5%). A decrease in the PUFA *n*-6 to *n*-3 ratio to 6.55:1 in mature milk was observed as lactation progressed. As reported in the literature, the ratio of PUFA *n*-6:*n*-3 in breast milk varies depending on the woman’s origin. According to published results, it was higher in Asian countries (China—12.5:1; Malaysia—10.1:1) than in North America (Canada—6.5:1; United States—7.6:1) [6]. The average PUFA *n*-6:*n*-3 ratio obtained in the study for Polish women (6.59:1) is similar to the average ratio (6.53 ± 1.72:1) reported for 55 countries worldwide [2]. The lower PUFA *n*-6:*n*-3 ratio indicates a higher intake of oily marine fish and fish oil supplements during pregnancy and lactation, and thus the formation of higher PUFA *n*-3 levels in milk [26]. In contrast, Nishmura et al. [78] and Silva et al. [79] suggest that the high consumption of vegetable oil (especially soybean oil) promotes a higher PUFA *n*-6:*n*-3 ratio, reaching values above 10:1. According to data from the Food and Nutrition Institute, fish consumption in Poland is at a low level in contrast to other European countries [80]. The European Market Observatory for Fisheries and Aquaculture (EUMOFA) reports that despite the increasing trend of fish consumption in Poland, it is still low (14.26 kg/per capita/year) [81]. The mothers’ BMI is directly related to diet. Hua et al. [44] report that overweight and obese women were more likely to consume processed foods, which may cause modifications in fatty acid metabolism. This leads to the occurrence of reduced PUFA *n*-3 levels, elevated PUFA *n*-6 levels, and atrophic changes in the PUFA *n*-6:*n*-3 fatty acid ratio.

The level of TFAs in breast milk is largely justified by the diet of nursing mothers, and therefore there is no specific level of occurrence of this group of acids. In this case, colostrum contained the most TFAs (3.86%), and their content significantly decreased in transition and mature milk by about 40%. Bousset-Alféres et al. [30] also noted a decline in total TFA content as lactation progressed. Since most of the available studies on TFA content in breast milk focus on mature milk, it is difficult to compare colostrum with mature milk. Studies have identified higher amounts of TFA in colostrum in female residents of Brazil (3.86% vs. 2.46%) [65] and also in Spain (3.86% vs. 0.45%) [32]. Similar results were found for mature milk—TFA content was higher than among women from Canada (2.28% vs. 1.90%) [82], China (2.28% vs. 0.77%) [83], and the United States (2.28% vs. 1.09%) [84]. However, women living in Croatia or Chilehad more TFAs in mature milk than milk from surveyed Polish women (2.28% vs. 2.30%; 2.28% vs. 3.28%,respectively) [65,85]. TFAs do not undergo de novo synthesis in the human body; therefore, their only source is dietary food products. A diet rich in dairy and meat products correlates primarily with increased amounts of VA and RA in breast milk. On the other hand, the intake of products containing hydrogenated vegetable oils leads to a higher proportion of TFAs (petroselaidic, elaidic) from industrial sources. The total TFA content of breast milk is affected by both the intake of TFAs from dairy and meat products and those from hydrogenated oils [86]. Mojska et al. [87] report that among Polish mothers, consumption of only bakery and confectionery products containing substantial amounts of industrially produced TFAs significantly increased the total amount of TFAs in breast milk. The source of TFAs in the diet of lactating women is of major importance. Excess TFAs from industrial sources can negatively affect the conversion of LA and ALA into AA and DHA. In contrast, VA and RA can modulate the infant’s immune system and are associated with a lower risk of allergies [88]. 

The ratios of individual PUFA *n*-3 to PUFA *n*-6 fatty acids are important in the composition of breast milk. DHA levels in breast milk were found to have a positive correlation with the intelligence quotient. In contrast, a negative correlation has been found between LA levels and the intelligence quotient. Consequently, one of the important fatty acid ratios in breast milk is the relationship between DHA and LA [51]. The ratios of these acids obtained in this study (0.05:1—C and TM; 0.04:1—MM) reached slightly higher values than those obtained by Aumeistere et al. [39] (0.03:1) and Lassek and Gaulin [89] (0.027:1). Another important post-experimental function is the balance between DHA and AA, which fluctuates between 0.70–0.79:1 in this case. Milk from Korean women had a higher ratio of DHA:AA (1.30:1), while milk from Pakistani women had a two times lower ratio of these acids (0.42:1) [90]. A decrease in the LA to ALA ratio was observed as the milk matured. The values recommended by the Nutrition Committee of ESPGHAN (European Society for Paediatric Gastroenterology, Hepatology, and Nutrition) for the ratio of these acids are 5–15:1, indicating that the determined values are within international recommendations [91,92]. Therefore, the ratio of LA to ALA acids obtained indicates the correct proportion of these acids in the diet of the women studied. Because of the low ALA content in the milk of Pakistani women, the ratio of LA:ALA in their milk was 35:1.

The composition of breast milk is so changeable that the fatty acid content can fluctuate within the same country. The average SFA content in the milk of Polish women from the Warmia and Mazury region obtained in our study was 50.58%, while Ćwiek et al. [93] report that in the West Pomeranian region of Poland, the average content of SFA in women’s milk is about 40%. On the other hand, in the Pomeranian region, women’s milk contained about 42% SFAs throughout lactation (from the 2nd to the 90th day of lactation) [94]. A similar content of SFAs was reported among women living in the region of central Poland (Mazovia Province) [53]. In connection with the higher level of SFAs in the milk of women in the Warmia and Mazury region, a correspondingly lower level of PUFAs (10.56%) was noted compared with milk from women from the West Pomeranian (17.64%) [93], Pomeranian (11.67%—averaged content from 2 to 90 days of lactation) [94], and Mazovian (15.13%) regions [53]. Differences in PUFA levels in breast milk within different regions of Poland may be explained by different feeding styles. Based on Polish statistics from the Social Surveys Department in 2018 [95], fish consumption in the West Pomeranian region was the highest in Poland (0.33 kg/per capita/month) and exceeded the national average consumption (0.28 kg/per capita/month).

#### 4.2.2. Infant Formulas

The infant formulas showed the same fatty acid proportion: SFA > MUFA > PUFA. The varying levels of MUFAs observed in infant formulas may be related to the different oils and fats (e.g., canola, coconut) and their proportions added at the production stage [25]. A similar situation was observed for the PUFA content, where IF-I contained higher amounts of *n*-6 PUFAs. Despite the presence of the same proportion of fatty acids in both types of infant formulas (SFA > MUFA > PUFA), a particular variability in the PUFA profile was reported, which may be mainly caused by the different sources of fat used in the production of each infant formula [28]. Increasingly, oils derived from unconventional sources, such as single-celled organisms, are being used as ingredients in infant formula. Among these oils, the most common are those extracted from *Mortierella alpina* or *Schizochytrium* sp., which are distinguished by their high content of PUFA *n*-3 and PUFA *n*-6 [25]. The TFA content of infant formulas was in line with The European Society for Paediatric Gastroenterology, Hepatology, and Nutrition (ESPGHAN) recommendations for infant formulas (addition of no more than 3% of total fatty acids) [96]. The small amounts of TFAs in infant formula are a result of the presence of these acids in cow’s milk, from which the tested products were produced.

Based on calculations of the content of each fatty acid group (SFA, MUFA, PUFA) declared on the manufacturer’s label, the SFA content was the most similar to the results of our tests. The manufacturer declared the presence of SFAs at the level of 43.65% and 44.60% in IF-I and IF-II, while in our research, these values were 45.07% and 44.07%. It should be noted that the reported SFA values determined by the authors take into account BCFAs—*iso* 15, *anteiso* 15, *iso* 16—which are included in SFAs [57]. Slightly larger deviations were observed in the sum of MUFAs. According to the manufacturer, the sum of these acids in IF-I and IF-II was 39.68% and 39.57%, respectively, while the authors showed the presence of these acids in amounts of 38.11% and 35.62% after relativizing the geometric isomers of monounsaturated fatty acids present, taking into account a double bond in the trans configuration (C18:1 *n*6+*n*9 *t*) [97]. In this case, 4% lower amounts of MUFAs were identified in IF-II than that declared by the manufacturer. A similar situation was observed in IF-I in the total PUFAs reported by the manufacturer (16.67%) and determined by the authors (15.84%). This may be due to the specificity of the equipment and the accuracy of the method, as well as to the fact that selected MUFA and PUFA acids were not identified. Some researchers have identified in infant formulas acids such as nervonic (C24:1 *n*-9), γ-linolenic (C18:3 *n*-6), and dihomo-γ-linolenic acid (C20:3 *n*-6), [28,98], which were not identified in the study conducted. These acids may have been present in the infant formulas tested, slightly affecting the differences in the content of claimed and labeled MUFAs and PUFAs.

As mentioned previously, the composition of PUFAs in infant formulas depends on the type of oils they contain, which are added during their manufacture. Due to the different ratios of LA and ALA, the obtained ratio of these acids in IFs was also staggered (6:05:1 vs. 10.72:1). Both counted ratios met established standards. To ensure that infant formulas are most similar to human milk, manufacturers must use appropriate mixes of vegetable oils containing LA and ALA acids to reach an appropriate ratio in the range of 5:1 to 15:1 [99]. In current infant formula composition recommendations, it is stated that high LA/ALA ratios should be avoided due to the reduced conversion of ALA to *n*-3 LCPUFA. In addition, the presence of excessively high LA levels in IFs may promote the transformation of LA to AA and oxidized lipids with pro-inflammatory functions, which may adversely influence immunity and the development of infants’ cognitive and metabolic functions [100]. The ratio of DHA:AA acids was 0.81:1 and 0.92 in IF-I and IF-II, respectively. According to Codex Alimentarius recommendations [56], the addition of AA in infant formulas should occur in a concentration at least equal to that of DHA, and the infant formulas tested met these standards. It is crucial to maintain the appropriate ratio of DHA and AA, as the supply of only one can lead to negative health effects in infants, limiting proper growth and affecting the immune system [55]. The DHA:LA ratio in infant formulas was similar to those obtained in mature breast milk. Based on the results of Chen et al. [27], the DHA:LA ratio was calculated, and the values obtained were lower than in our study (0.01:1–0.02:1).

### 4.3. Associations between Obtained Data—Multivariate Analysis (Principal Component Analysis and Cluster Analysis)

The use of PCA is designed to reduce the original set of variables to the smallest set of uncorrelated components, which represents most of the information found in the original variables. With a larger number of variables, this method enables effective interpretation of the relationships within the data [101]. The position of cases in the plot is an indication of the different fatty acid profiles of breast milk fractions. The available data allow the grouping and differentiation of fatty acid profiles of individual milk from each other. Colostrum differed in composition relative to transitional and mature milk, as evidenced by the opposite position of cases on the plot. Cases of infant formulas were on the opposite side of the score plot, as evidenced by their different fatty acid profiles relative to human milk. The difference in colostrum is largely determined by the higher presence of VA and AA. According to the study, VA and AA levels were highest in colostrum. The different profiles in infant formulas were largely determined by higher amounts of C18:1 *n*-9 (IF-I) and C18:2 *n*-6 (IF-II), as well as TFAs and BCFAs. 

The use of CA is to arrange objects into groups in a way that shows the degree of association of objects from the same or different groups [102]. The dendrogram shows the mutual similarity and differences in individual milk samples with regard to the fatty acids present. Similarly, as described in the results section, the fractions of breast milk varied, with colostrum differing most significantly. This confirms that the lactation period has a significant effect on the fatty acid profile. On the other hand, infant formulas were on the other side of the cluster on the dendrogram, as evidenced by their different lipid profiles relative to human milk.

Multivariate methods are an effective tool for studying the relationship between the fatty composition of different fractions of breast milk and infant formulas, as also presented by other authors [5,10,103].

### 4.4. Limitations and Strengths of the Study

A limitation of the present study is the lack of analysis of the current/habitual dietary intake of lactating women. Because more than 50% of the women surveyed provided incomplete (or no) data on their current intake, the authors were unable to undertake an analysis of the correlation between the fatty acid profile and the diet of nursing mothers. Similarly, in the case of medications or dietary supplements taken, the women did not provide complete data on the type of preparation and the proportion of each ingredient. Such information would be valuable for analyzing the effects of individual medications/dietary supplements on fatty acid levels. In particular, medications or supplements with added DHA or EPA could significantly affect the levels of these acids in breast milk. In addition, the specific time of milk sampling was not specified, because of the on-demand feeding of children. Out of concern for the comfort of mother and child, women were asked to collect milk at a convenient time, after feeding their child. Another limitation is the size of the group of women (*n* = 69) with milk samples analyzed. A larger number of milk samples analyzed would have increased the representativeness of the results obtained.

A strength of this study is the analysis of fatty acid composition among women living in the Warmia and Mazury region. There are studies available in the literature on the fatty acid profile of Polish women from the West Pomeranian Region of Poland [93], the Pomeranian Region [94], or the Mazovia region [53]. Although some authors have studied the milk of mothers from the north-eastern region of Poland, there is a gap in the literature regarding the formation of specific fatty acid groups concerning lactation periods. In addition, the work is distinguished by the use of multivariate statistics, which allowed the determination of which fatty acids contribute most to the different fatty acid profiles between maternal milk from different lactation periods, between selected infant formulas, and between breast milk and infant formulas.

The authors plan to continue their research on the breast milk lipidome. In future studies, the authors plan to focus on analyzing the food intake of breastfeeding women and to reliably assess the impact of their habitual diet on the breast milk lipidome. In addition, given the limitations in the literature regarding the fatty acid profile of specialty infant formulas (including foods for special infant use), the authors intend to analyze these products to complete the data on the lipid profile of infant formulas.

## 5. Conclusions

The present research was designed to evaluate the variability in the fatty acids in the milk of women from the Warmia and Mazury region from different periods of lactation. The results were then compared with selected formulas for follow-on infant feeding. The predominant fatty acids in breast milk were palmitic acid (about 28%), oleic acid (about 30%), and linoleic acid (about 8.60%). Of the fatty acids, those that were lactation-dependent were C8:0, C10:0, C12:0, and C14:0, which increased as lactation progressed, and C22:1 *n*-9, C20:4 *n*-6, and C22:6 *n*-3, which decreased as the milk matured (*p* < 0.05). The average ratio of PUFA *n*-6 to PUFA *n*-3 acids for the three lactation periods was 6.59:1, similar to the global average ratio of 6.53 ± 1.72:1. The fatty acid composition of the infant formulas differed, especially in containing higher amounts of PUFA *n*-3 PUFA *n*-6, and TFAs and lower BCFAs. Principal component analysis showed that colostrum differed the most from transitional and mature milk, with higher amounts of vaccenic and arachidonic acid. In contrast, infant formula samples differ in composition from human milk primarily due to significantly lower levels of TFAs and BCFAs.

In further research, it will be important to investigate the influence of current or habitual food consumption on the formation of the fatty acid profile in the milk of mothers from the Warmia and Mazury region. In addition, it is planned to analyze more infant formulas, taking into account those intended for specialized infant nutrition.

## Figures and Tables

**Table 1:** The nutritional value of the infant formulas studied.

Nutritional Value in 100 g of Powder *	IF-I	IF-II
Energy (kcal)	482	511
Fat (g), of which	25	27.8
Saturated fatty acids (g)	11	12.4
Monounsaturated fatty acids (g)	10	11
Polyunsaturated fatty acids (g)	4.2	4.4
Carbohydrates (g), of which	51	54.3
Sugars (g)	24	23.1
Protein (g)	11	9.8
Preparation of 100 mL of milk	13.8 g of powder + 90 mL of water	12.8 g of powder + 100 mL of water

Abbreviations: IF-I, IF-II—infant formulas from different manufacturers. * Data were obtained from the labels of individual infant formulas.

**Table 2:** Content of SFAs, MUFAs, and PUFAs in 100 mL of tested ready-to-eat infant formulas and as % of total fatty acids.

Group of Fatty Acids	IF-I		IF-II	
	In 100 mL of the Prepared Milk *	% of Total Fatty Acids	In 100 mL of Prepared Milk	% of Total Fatty Acids
Saturated fatty acids	1.52	43.65	1.59	44.60
Monounsaturated fatty acids	1.38	39.68	1.41	39.57
Polyunsaturated fatty acids	0.58	16.67	0.56	15.83

* Calculations were made based on data from Table 1, using information for the preparation of 100 mL of infant formula. Abbreviations: IF-I, IF-II—infant formulas from different manufacturers.

**Table 3:** Content of saturated fatty acids (SFAs), monounsaturated fatty acids (MUFAs), polyunsaturated *n*-3 and *n*-6 fatty acids (PUFAs *n*-3 and *n*-6), branched-chain fatty acids (BCFAs) and trans fatty acids (TFAs) in human milk according to lactation period and in selected infant formulas. The total SFA, MUFA, and PUFA content and the ratio of selected fatty acids in breast milk and infant formulas (%) are shown.

Fatty Acid	Common Name	Colostrum	Transitional Milk	Mature Milk	IF-I	IF-II
C6:0	Caproic	ND	0.03 ± 0.05 ^b^	0.01 ± 0.05 ^b^	0.21 ± 0.02 ^a^	0.17 ± 0.00 ^a^
C8:0	Caprylic	0.08 ± 0.06 ^c^	0.26 ± 0.19 ^b^	0.33 ± 0.32 ^b^	2.25 ± 0.20 ^a^	1.78 ± 0.00 ^a^
C10:0	Capric	0.92 ± 0.40 ^b^	1.65 ± 0.63 ^a^	2.06 ± 1.04 ^a^	1.77 ± 0.13 ^a^	1.40 ± 0.01 ^a^
C12:0	Lauric	3.99 ± 1.69 ^c^	5.99 ± 2.94 ^b^	6.09 ± 2.30 ^b^	12.95 ± 0.41 ^a^	10.26 ± 0.06 ^a^
C14:0	Myristic	5.94 ± 1.52 ^b^	7.43 ± 2.47 ^b^	9.21 ± 2.22 ^a^	5.38 ± 0.05 ^b^	4.47 ± 0.02 ^b^
C15:0	Pentadecylic	0.36 ± 0.17 ^a^	0.40 ± 0.17 ^a^	0.40 ± 0.18 ^a^	0.06 ± 0.00 ^b^	0.04 ± 0.00 ^b^
C16:0	Palmitic	28.10 ± 6.06 ^a^	29.24 ± 6.14 ^a^	26.58 ± 8.75 ^a^	18.82 ± 0.17 ^a^	21.68 ± 1.44 ^a^
C17:0	Margaric	0.36 ± 0.11 ^a^	0.39 ± 0.11 ^a^	0.38 ± 0.15 ^a^	0.07 ± 0.00 ^b^	0.09 ± 0.00 ^b^
C18:0	Stearic	6.72 ± 1.55 ^a^	7.13 ± 2.03 ^a^	7.58 ± 2.01 ^a^	3.17 ± 0.02 ^b^	3.88 ± 0.10 ^b^
C20:0	Arachidic	0.03 ± 0.08 ^b^	0.02 ± 0.04 ^b^	0.07 ± 0.09 ^b^	0.33 ± 0.00 ^a^	0.29 ± 0.01 ^a^
C14:1 *n*5	Myristoleic	0.14 ± 0.10 ^a^	0.17 ± 0.06 ^a^	0.17 ± 0.09 ^a^	0.02 ± 0.00 ^b^	0.03 ± 0.02 ^b^
C16:1 *n*7	Palmitoleic	1.90 ± 0.66 ^a^	2.43 ± 3.53 ^a^	1.89 ± 0.76 ^a^	0.20 ± 0.01 ^b^	0.16 ± 0.00 ^b^
C17:1 *n*9	cis-10-Heptadecenoic acid	0.14 ± 0.03 ^a^	0.13 ± 0.03 ^a^	0.15 ± 0.05 ^a^	0.04 ± 0.01 ^b^	0.03 ± 0.00 ^b^
C18:1 *n*9	Oleic	31.46 ± 6.35 ^b^	30.05 ± 4.83 ^b^	29.18 ± 8.19 ^b^	37.34 ± 0.14 ^a^	35.10 ± 0.20 ^a^
C20:1 *n*9	Eicosenoic	0.46 ± 0.36 ^a^	0.40 ± 0.36 ^a^	0.39 ± 0.25 ^a^	0.43 ± 0.00 ^a^	0.15 ± 0.01 ^b^
C22:1 *n*9	Ercucic	0.14 ± 0.04 ^a^	0.09 ± 0.16 ^b^	0.10 ± 0.19 ^b^	ND	ND
C18:2 *n*6	Linoleic (LA)	8.49 ± 1.77 ^b^	8.59 ± 1.86 ^b^	8.83 ± 2.58 ^b^	12.76 ± 0.21 ^a^	16.62 ± 0.07 ^a^
C20:4 *n*6	Arachidonic (AA)	0.61 ± 0.18 ^a^	0.52 ± 0.22 ^b^	0.47 ± 0.28 ^b^	0.52 ± 0.11 ^b^	0.49 ± 0.07 ^b^
C18:3 *n*3	α-linoleic (ALA)	0.72 ± 0.52 ^c^	0.84 ± 0.69 ^c^	0.94 ± 0.60 ^c^	2.11 ± 0.03 ^a^	1.55 ± 0.01 ^b^
C20:5 *n*3	Eicosapentaenoic (EPA)	0.17 ± 0.21 ^a^	0.18 ± 0.26 ^a^	0.15 ± 0.28 ^a^	0.03 ± 0.01 ^b^	0.04 ± 0.02 ^b^
C22:6 *n*3	Docosahexaenoic (DHA)	0.44 ± 0.14 ^a^	0.41 ± 0.04 ^b^	0.33 ± 0.06 ^b^	0.42 ± 0.10 ^a^	0.45 ± 0.07 ^a^
*iso* C15:0	Isopentadecylic	0.06 ± 0.03 ^a^	0.07 ± 0.03 ^a^	0.07 ± 0.04 ^a^	ND	ND
*anteiso* C15:0	Anteisopentadecylic	0.10 ± 0.07 ^a^	0.12 ± 0.06 ^a^	0.10 ± 0.07 ^a^	ND	0.01 ± 0.00 ^b^
*iso* C16:0	Isopalmitic	0.07 ± 0.05 ^a^	0.09 ± 0.05 ^a^	0.08 ± 0.04 ^a^	0.06 ± 0.00 ^a^	ND
C18:1 *n*6+*n*9 *t*	Petroselaidic and Elaidic	0.56 ± 0.29 ^a^	0.45 ± 0.23 ^a^	0.49 ± 0.25 ^a^	0.08 ± 0.02 ^b^	0.15 ± 0.02 ^b^
C18:1 *t*11	Vaccenic (VA)	2.99 ± 4.19 ^a^	1.74 ± 0.98 ^a^	1.52 ± 1.26 ^a^	ND	ND
C18:2 *c*9*t*11	Rumenic (RA)	0.21 ± 0.43 ^a^	0.13 ± 0.11 ^b^	0.18 ± 0.29 ^a^	ND	ND
C16:1 *t*7	Palmitelaidic	0.10 ± 0.05 ^a^	0.10 ± 0.06 ^a^	0.09 ± 0.07 ^a^	ND	ND
SFAs		46.50 ^b^	52.54 ^a^	52.71 ^a^	45.01 ^b^	44.06 ^b^
MUFAs		34.24 ^b^	33.27 ^b^	31.88 ^c^	38.03 ^a^	35.47 ^b^
PUFAs; *n*6		9.10 ^c^	9.11 ^c^	9.30 ^c^	13.28 ^b^	17.11 ^a^
PUFAs; *n*3		1.33 ^c^	1.43 ^c^	1.42 ^c^	2.56 ^a^	2.04 ^b^
BCFAs		0.23 ^a^	0.28 ^a^	0.25 ^a^	0.06 ^b^	0.01 ^c^
TFAs		3.86 ^a^	2.42 ^b^	2.28 ^b^	0.08 ^c^	0.15 ^c^
PUFA *n*6:PUFA *n*3		6.84:1	6.37:1	6.55:1	5.19:1	8.39:1
DHA:LA		0.05:1	0.05:1	0.04:1	0.03:1	0.03:1
DHA:AA		0.72:1	0.79:1	0.70:1	0.81:1	0.92:1
LA:ALA		11.79:1	10.23:1	9.39:1	6.05:1	10.72:1

Abbreviations: IF-I, IF-II—infant formulas from different manufacturers, SFAs—saturated fatty acids, MUFAs—monounsaturated fatty acids, PUFAs—polyunsaturated fatty acids, BCFAs—branched-chain fatty acids, TFAs—trans fatty acids, DHA—docosahexaenoic acid, LA—linoleic acid, AA—arachidonic acid, ALA—α-linoleic acid, ND—non detected. Means with different letters (a, b, c) are significantly different at *p* < 0.05. Means a, b, c specify differences between fatty acid composition in human milk depending on the lactation period and between breast milk from different lactation periods and infant formulas from two producers.

## Data Availability

Data is contained within the article and Supplementary Materials.

## Supplementary Materials

The following supporting information can be downloaded at: https://www.mdpi.com/article/10.3390/nu16152411/s1, Table S1: Characteristics of the women studied.

## Abbreviations

AA	arachidonic acid
ALA	α-linoleic acid
BCFA	branched-chain fatty acid
C	colostrum
C10:0	capric acid
C12:0	lauric acid
C14:0	myristic acid
C14:1 *n*-5	myristoleic acid
C15:0	pentadecylic acid
C16:0	palmitic acid
C16:1 *n*-7	palmitoleic acid
C16:1 *t*7	palmitelaidic acid
C17:0	margaric acid
C18:0	stearic acid
C18:1 *n*6+*n*9 *t*	petroselaidic and elaidic acids
C18:1 *n*-9	oleic acid
C18:1 *t*11	vaccenic acid
C18:2 *c*9*t*11	rumenic acid
C18:2 *n*-6	linoleic acid
C18:3 *n*-3	α-linoleic acid
C20:0	arachidic acid
C20:1 *n*-9	eicosenoic acid
C20:4 *n*-6	arachidonic acid
C20:5 *n*-3	eicosapentaenoic acid
C22:1 *n*-9	ercucic acid
C22:6 *n*-3	docosahexaenoic acid
C6:0	caproic acid
C8:0	caprylic acid
CA	cluster analysis
CLA	conjugated linoleic acid
DHA	docosahexaenoic acid
EPA	eicosapentaenoic acid
HM	human milk
IF	infant formula
*iso* C15:0	isopentadecylic acid
*iso* C16:0	isopalmitic acid
LA	linoleic acid
LCPUFA	long-chain polyunsaturated fatty acid
MCFA	medium-chain fatty acid
MM	mature milk
MUFA	monounsaturated fatty acid
PCA	principal component analysis
PUFA *n*-3	polyunsaturated fatty acid *n*-3
PUFA *n*-6	polyunsaturated fatty acid *n*-6
RA	rumenic acid
SCFA	short-chain fatty acid
SFA	saturated fatty acid
TFA	trans fatty acid
TM	transitional milk
VA	vaccenic acid
