# Supplementary material for: Changes in the Fatty Acid Profile of Lactating Women Living in Poland—A Comparison with the Fatty Acid Profile of Selected Infant Formulas
Source: Nutrients. 2024 Jul 25;16(15):2411. doi: 10.3390/nu16152411 (PMC11314165; doi:10.3390/nu16152411)
Supplement: Supplementary file 1 [file nutrients-16-02411-s001.zip › nutrients-3101703-supplementary.pdf]

Table S1. Characteristic of the women studied.

Factors	Mean±SD
Age, years	30.69±6.38
Body weight, kg	67.64±9.05
Body height, m	1.65±0.06
BMI, kg/m^2	24.99±3.20
Characteristic	*n*=69
Characteristics of BMI	
Normal body weight	*n*=31
Overweight and obese	*n*=38
Stage of lactation	
1	*n*=21
2	*n*=26
3	*n*=22
